# Supplementary material for: Resource and location sharing in wireless networks
Source: PLoS One. 2024 Feb 23;19(2):e0299396. doi: 10.1371/journal.pone.0299396 (PMC10889630; doi:10.1371/journal.pone.0299396)
Supplement: Attachment — Submitted filename: review.pdf [file pone.0299396.s001.pdf]

Review of manuscript PONE-D-23-32266

This paper studied the resource sharing problem in the context of base station (BS) co-location in wireless networks with multiple service operators. More specifically, assuming a given co-location overlapping factor, the authors define a new metric called "link strength" and derived the expected value of the link strength as a function of the overlapping factor and found the optimal overlapping factor. The paper is well written. However, I have the following concerns regarding the technical contributions.

1. The Poisson point process (PPP) is used to model the stochastic distribution of the BSs and users. In PPP, there is no minimum distance separation, i.e., points can be arbitrarily close to each other. This is not true in practice—the is usually a minimum distance between BSs (say at least 200 meters apart from each other). This minimum distance can have significant impact on the network capacity analysis. It would be better to consider such more practical models (I am not sure if this is doable mathematically).
2. Page 4, line 114, the overlapping factor p has not been rigorously defined.
3. Page 5, line 120, what is the "degree" of a BS? Is it the number of users that are connected to this BS?
4. Regarding the channel model (eq. (4)), it seemed that users of the same BS are allocate nonoverlapping sub-bands so they do not interfere with each other. Therefore, SNR instead of SINR is used. The channel model should be clearly described. In practice, inter-BS interference exists which seems to be omitted in the channel model. How do you justify this simplification?
5. What is the rationale behind the definition of the utility metric "user strength"? To enforce fairness among users (i.e., users with low throughput should be prioritized a little bit such they can get adequate service), a concave utility function (say logarithm function) is typically used on top of the Shannon throughput. However, the utility definition is a bit counterintuitive as it takes the SNR out of the logarithm of Shannon capacity. How do you justify the throughput performance under this utility function?
6. In Fig. 3, the authors claimed that the average channel capacity is a decreasing function of r, then why is the first point with a very small r having a very low capacity? In addition, the power decay factor alpha is set to be 1 which is not practical at all—it is known that in a free space radiation model, the power decays at least quadratically with the distance. In practical scenarios alpha can only be larger. It seemed that in eq (5) it is also assumed a decay factor of 1.

7. An upper bound on p has been derived in eq (18) and a comparison between this bound and the true values is shown in Fig. 6. In Fig. 6, for a given beta_2, why is that the upper bound is smaller than the true values?

Minor issues and typos:

1. In Fig. 6, part of the x-axis label is cut off which should be corrected.
2.
