# Supplementary material for: Resource and location sharing in wireless networks
Source: PLoS One. 2024 Feb 23;19(2):e0299396. doi: 10.1371/journal.pone.0299396 (PMC10889630; doi:10.1371/journal.pone.0299396)
Supplement: Attachment — Submitted filename: Response to Reviewers.pdf [file pone.0299396.s002.pdf]

Reply to the Reviewers

Re: Manuscript ID D-23-32266
"Resource and location sharing in wireless networks"
Clara Stegehuis, Lotte Weedage
PLOS ONE

Reviewer #1, comment #1

The Poisson point process (PPP) is used to model the stochastic distribution of the BSs and users. In PPP, there is no minimum distance separation, i.e., points can be arbitrarily close to each other. This is not true in practice—there is usually a minimum distance between BSs (say at least 200 meters apart from each other). This minimum distance can have significant impact on the network capacity analysis. It would be better to consider such more practical models (I am not sure if this is doable mathematically).

Our response #1.1

Indeed, in practice the Poisson point process does not fully characterize the distribution of BSs in a plane, as BSs are in practice not arbitrarily close to each other. However, still several works show that the PPP assumption is reasonable on a large scale (see for example (1)). This, combined with its mathematical tractability was the main reason to have a PPP as underlying model.

(1) Błaszczyszyn, Bartłomiej, Mohamed Kadhem Karray, and Holger Paul Keeler. "Using Poisson processes to model lattice cellular networks." 2013 Proceedings IEEE INFOCOM. IEEE, 2013.

Reviewer #1, comment #2

Page 4, line 114, the overlapping factor p has not been rigorously defined.

Our response #1.2

We have clarified the definition of p and provided an example to make the definition of the co-location factor p more clear in the revised manuscript.

Reviewer #1, comment #3

Page 5, line 120, what is the "degree" of a BS? Is it the number of users that are connected to this BS?

Our response #1.3

Indeed, we define the degree of a BS as the number of users that are connected to this BS and we have clarified this in our updated manuscript.

Reviewer #1, comment #4

Regarding the channel model (eq. (4)), it seemed that users of the same BS are allocate non-overlapping sub-bands so they do not interfere with each other. Therefore, SNR instead of SINR is used. The channel model should be clearly described. In practice, inter-BS interference exists which seems to be omitted in the channel model. How do you justify this simplification?

Our response #1.4

In the revised manuscript, we added an explanation on why we omit interference in this system model in the Section "Link strength analysis", page 5.

Reviewer #1, comment #5

What is the rationale behind the definition of the utility metric "user strength"? To enforce fairness among users (i.e., users with low throughput should be prioritized a little bit such they can get adequate service), a concave utility function (say logarithm function) is typically used on top of the Shannon throughput. However, the utility definition is a bit counter intuitive as it takes the SNR out of the logarithm of Shannon capacity. How do you justify the throughput performance under this utility function?

Our response #1.5

We chose to use this utility metric to ensure more fairness between users. Indeed, this metric is similar to a the logarithm of the Shannon throughput but then with the SNR out of the logarithm to make it possible to find exact expressions of the utility function. For small values of the SNR, these two quantities are similar, since $\log(1+x) = x + O(x^2)$. We clarified this in the revised manuscript.

Reviewer #1, comment #6

In Fig. 3, the authors claimed that the average channel capacity is a decreasing function of r, then why is the first point with a very small r having a very low capacity? In addition, the power decay factor alpha is set to be 1 which is not practical at all—it is known that in a free space radiation model, the power decays at least quadratically with the distance. In practical scenarios alpha can only be larger. It seemed that in eq (5) it is also assumed a decay factor of 1.

Our response #1.6

Indeed, we have assumed that the path loss factor α is equal to 1 in this manuscript, while in practice the decay factor will be larger. We have changed Figure 3 to reflect a scenario with $\alpha = 2$, but for the rest of the manuscript assume that $\alpha = 1$ for simplified notation. Since this path loss factor only contributes as a constant in the utility function due to the logarithm, another value of α will not change any analyses.

Moreover, the reviewer is right that in Figure 3 the channel capacity first increases in r: this is because for very small values of r, only very few users are connected which automatically results in a low average channel capacity. Thus, r should be sufficiently large such that at least some users receive a connection (at least one user per BS). We have clarified this in the revised manuscript.

Reviewer #1, comment #7

An upper bound on p has been derived in eq (18) and a comparison between this bound and the true values is shown in Fig. 6. In Fig. 6, for a given β_2, why is that the upper bound is smaller than the true values?

Our response #1.7

This was unclear indeed, and we have clarified this in our revised manuscript. The upper bound in Figures 6 and 9 is an approximation of the real upper bound (denoted as 'real value'). Thus, the blue line does not denote an upper bound of the purple dots, but they both represent the same upper bound on when it will be beneficial to use location sharing or not. We have changed the labels in Figure 6 and 9 to clarify this.
